# School attendance and sport participation amongst children with chronic kidney disease: a cross-sectional analysis from the Kids with CKD (KCAD) study

**DOI:** 10.1007/s00467-023-06198-0

**Published:** 2023-11-09

**Authors:** Adam C. Hudson, Anita van Zwieten, Kylie-Ann Mallitt, Anne Durkan, Deirdre Hahn, Chandana Guha, Rabia Khalid, Madeleine Didsbury, Anna Francis, Steven McTaggart, Fiona E. Mackie, Chanel Prestidge, Armando Teixeira-Pinto, Suncica Lah, Martin Howell, Kirsten Howard, Natasha Nassar, Allison Jaure, Jonathan C. Craig, Germaine Wong, Siah Kim

**Affiliations:** 1 Centre for Kidney Research, The Children’s Hospital at Westmead, Cnr Hainsworth St and Hawkesbury Road, Westmead, NSW 2145 Australia; 2 Sydney School of Public Health, The University of Sydney, Sydney, Australia; 3 Department of Nephrology, The Children’s Hospital at Westmead, Westmead, Australia; 4 Child & Adolescent Renal Service, Queensland Children’s Hospital, Brisbane, Australia; 5 Department of Nephrology, Sydney Children’s Hospital at Randwick, Sydney, Australia; 6 Department of Nephrology, Starship Children’s Hospital, Auckland, New Zealand; 7 School of Psychology, The University of Sydney, Sydney, Australia; 8 Menzies Centre for Health Policy and Economics, Faculty of Medicine and Health, University of Sydney, Sydney, Australia; 9 Child Population and Translational Health Research, Children’s Hospital at Westmead Clinical School, Faculty of Medicine and Health, The University of Sydney, Sydney, Australia; 10 College of Medicine and Public Health, Flinders University, Adelaide, Australia; 11 Centre for Transplant and Renal Research, Westmead Hospital, Sydney, Australia

**Keywords:** Children, Adolescents, Chronic kidney disease, Kidney replacement therapy, Dialysis, Transplantation, Life participation, School, Sport

## Abstract

**Background:**

School attendance and life participation, particularly sport, is a high priority for children with chronic kidney disease (CKD). This study is aimed at assessing the association between CKD stage, sports participation, and school absences in children with CKD.

**Methods:**

Using data from the binational Kids with CKD study (ages 6–18 years, *n* = 377), we performed multivariable regression to evaluate the association between CKD stage, school absences, and sports participation.

**Results:**

Overall, 62% of participants played sport with the most frequent sport activities engaged in being swimming (17%) and soccer (17%). Compared to children with CKD 1–2, the incidence rate ratios (IRR) (95% CI) for sports participation amongst children with CKD 3–5, dialysis, or transplant were 0.84 (0.64–1.09), 0.59 (0.39–0.90), and 0.75 (0.58–0.96), respectively. The median (IQR) days of school absences within a four-week period were 1 day (0–1), with children on dialysis reporting the highest number of school absences (9 days (5–15)), followed by transplant recipients (2 days (1–7)), children with CKD 3–5 (1 day (0–3)), and with CKD 1–2 (1 day (0–3)). Duration of CKD modified the association between CKD stage and school absences, with children with a transplant experiencing a higher number of missed school days with increasing duration of CKD, but not in children with CKD 1–5 or on dialysis (*p*-interaction < 0.01).

**Conclusions:**

Children receiving dialysis and with a kidney transplant had greater school absences and played fewer sports compared to children with CKD stages 1–2. Innovative strategies to improve school attendance and sport participation are needed to improve life participation of children with CKD.

**Graphical abstract:**

**Figure:**
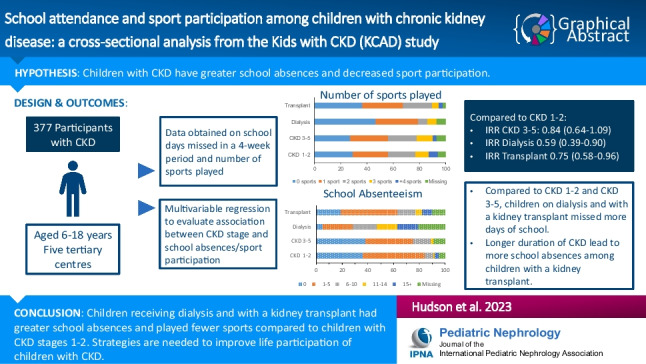
A higher resolution version of the Graphical abstract is available as Supplementary information

**Supplementary Information:**

The online version contains supplementary material available at 10.1007/s00467-023-06198-0.

## Introduction

Equitable access to learning is the cornerstone of achieving optimal educational attainment and overall well-being for all children, irrespective of their health status, and who they are and where they live. However, children with chronic kidney disease (CKD) suffer significant survival, quality of life, and developmental disadvantage because of their ill health. Children receiving kidney replacement therapy (KRT), the most advanced stage of CKD, have poorer cognition and academic achievement with mean full-scale intelligence quotient 10.5 lower than the general population [1] and such gaps worsen over time [2]. This in turn contributes to lower educational attainment and employment as adults [3, 4].

Given the pervasive impact of CKD across the life course, life participation has been identified as a core outcome for children with CKD, with being able to attend school, participate in sport and engaging in social activities with their peers identified as the key aspects of life participation considered by patients and their caregivers [5, 6]. Engagement in daily activities such as sports and schooling, that are typically enjoyed by children without chronic illness, is lower in children treated with dialysis [7]. Patients report that symptoms such as fatigue and pain restrict their daily activities, and the frequent infections, hospitalisation, and medical appointments have a cumulative effect over time, monopolising their lives and restricting their ability to attend school and participate in extra-curricular activities which contribute to poorer academic attainment, social isolation, and lower self-esteem [8]. The severity of CKD affects many aspects of life participation including school attendance and physical activity [9, 10], but no studies have quantified the extent to which reduced kidney function affects life achievements and participation, independent of confounding factors such as socioeconomic status (SES), age, and sex across the entire spectrum of CKD. The aims of this study were to quantify the number of missed school days and participation in sports in children with CKD. We also sought to determine the association between stage of CKD, sports participation, and school absences.

## Methods

This study was reported in accordance with the Strengthening the Reporting of Observational Studies in Epidemiology (STROBE) Guidelines [11].

### Study population and study design

This study used baseline cross-sectional data from the Kids with CKD study, a multi-centre longitudinal cohort study that was undertaken at five paediatric nephrology units across Australia and New Zealand, with recruitment occurring between 2012 and 2016. The study design has been previously described [12, 13]. Children aged 6–18 years with CKD stages 1–5 (CKD 1–5), on dialysis, or with kidney transplant were included. Participants were excluded if they were not receiving a formal education, were unable to provide written consent, or were from families where none spoke English. This study was approved by the Human Research Ethics Committees (HRECs) at participating centres: The Children’s Hospital at Westmead and Sydney Children’s Hospital (HREC/12/SCHN/159), Queensland Children’s Hospital (HREC/12/QCRH/113), the Royal Children’s Hospital (Royal Children’s Hospital Human Research Ethics Committee: 33229), and Starship Children’s Hospital (New Zealand Health and Disability Ethics Committees: 15/ NTB/37). Participants and/or their caregivers provided informed consent as appropriate for participant age.

### Outcomes

Information on sport participation and school attendance was obtained via self-reported questionnaires, which were completed by participants and/or their caregivers as appropriate to participant age, family situation, and preferences. Sport participation was assessed through the following questions: “Does your child do any sports?” and “If yes, please specify” with sports then listed as free text. The total number of types of sports played was categorised as follows: 0 sports played, 1 sport played, 2 sports played, 3 sports played, and ≥ 4 sports played. A descriptive analysis was undertaken to outline the most popular sports that our cohort played. School attendance was assessed by asking: “In the past 4 weeks, how many days was your child absent from school?”. The total number of school days absent over a four-week period (median interquartile range (IQR) was stratified by CKD stage and was adjusted to a ceiling of 20 days as this was the total number of school days in the preceding four weeks. Total number of school days absent over a four-week period was categorised as follows: 0 days missed, 1–5 days missed, 6–10 days missed, 11–14 days missed, and ≥ 15 days missed.

### Exposure

Exposure of the study was the CKD stage of the child (CKD 1–2, CKD 3–5, dialysis, and transplant). These data were obtained using the same questionnaire and cross-checked against medical records.

### Other covariates

The Kids with CKD study collected data on the causes of CKD, duration of CKD diagnosis, child and caregiver demographics, health insurance status, and family SES using a standardised questionnaire. A range of variables indicating SES were obtained by questionnaires, including caregiver educational attainment, household income, caregiver employment status, caregiver home ownership, and caregiver perceived financial status. All these parameters were combined using principal components analysis to calculate a global SES index score which was then used to define SES quartiles with the highest quartile reflecting the highest SES in the cohort, as previously described [12].

### Statistical analyses

Characteristics of the cohort were presented using descriptive statistics. Continuous variables were summarised as mean (standard deviation (SD)) if normally distributed, or median (interquartile range (IQR)) if not normally distributed. Categorical variables were presented as number (percentage). We investigated the association between CKD stage and total number of sports participated in using a multivariable Poisson regression, with a complete case analysis undertaken due to the small proportion of missing outcome and covariate data. To check the robustness of the results, we also fitted the models using negative binomial regressions. We considered the following covariates for inclusion: gender, causes of CKD, SES quartile, ethnicity, private health insurance, age of the participants at baseline, and duration of CKD diagnosis as these have been shown to be associated with school attendance and sport participation. Final covariates for the model were selected using a backward elimination approach, with variables being retained in the final model if they were statistically significant with *p* < 0.05 or were found to confound the effect of CKD stage by greater than 10%. We tested for effect modification between CKD stage and other variables in the final multivariable model with a *p* value of < 0.01 considered statistically significant. A similar statistical approach was taken to build the associative model between CKD stage and number of school days absent, with a multivariable negative binomial fitted using the same statistical parameters. The predicted number of days of school absent was extracted using marginal means. Analyses were undertaken in IBM SPSS Statistics 28.0 and Stata 17 BE.

## Results

### Baseline characteristics of the study participants

**Fig. 1:**
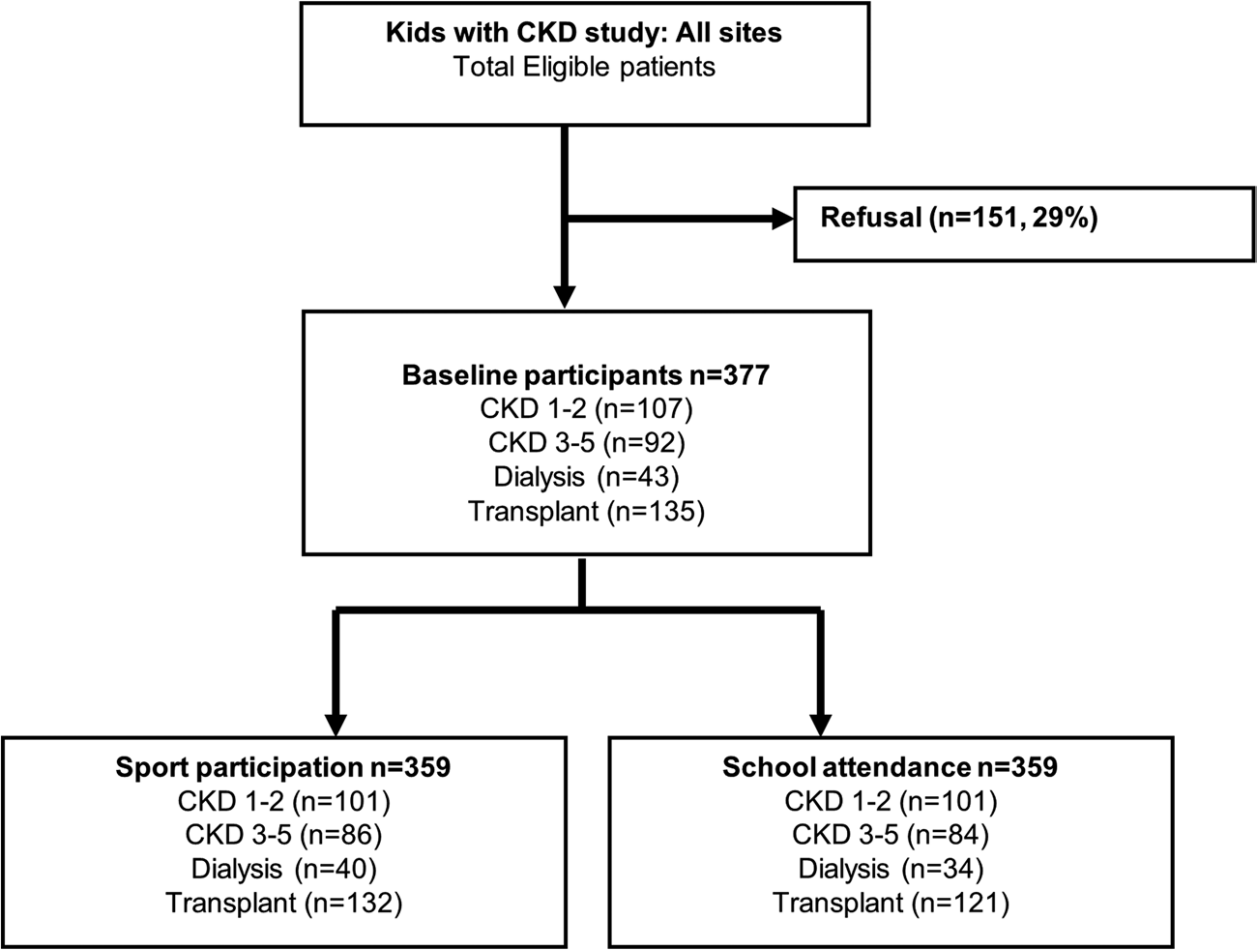
Study flow of Kids with CKD Study

Participant characteristics are outlined in Supplementary Table 1, and the selection of participants is shown in Fig. 1. Of 528 eligible participants, 377 consented and were included in the cohort. Of 377 participants, 107 (28%) had CKD 1–2, 92 (24%) had CKD 3–5, 43 (11%) were on dialysis, and 135 (36%) were transplant recipients. For school attendance, there were 36 (10%) missing responses (5 (5%) CKD 1–2, 8 (9%) CKD 3–5, 9 (21%) dialysis, and 14 (10%) transplant). Of the 36 participants (10%) who did not provide data on school attendance, five (14%) gave details for non-completion. The reasons included the children were on school holidays (*n* = 2), there were too many absences (*n* = 2), and one child has stopped school. Nine (out of 43) participants treated with maintenance dialysis (21%) did not provide any data on school absences, of which two (out of 9) participants reported that the number of school absences were very high and they were unable to quantity accurately. For sport participation, there were 18 (5%) participants with missing data (6 (6%) CKD 1–2, 6 (7%) CKD 3–5, 3 (7%) dialysis, and 3 (2%) transplant). Overall, 233 (62%) of participants were male. The mean (SD) age of the cohort was 12.2 years (3.8), with CAKUT (34%) being the major cause of CKD, followed by nephrotic syndrome (24%), glomerulonephritis (15%), and cystic kidney disease (8%). The majority of the study population were of European ethnicity (58%), followed by Asian (15%), and Middle Eastern (11%) ethnicities.

### Association between CKD stage and sports participation

**Fig. 2:**
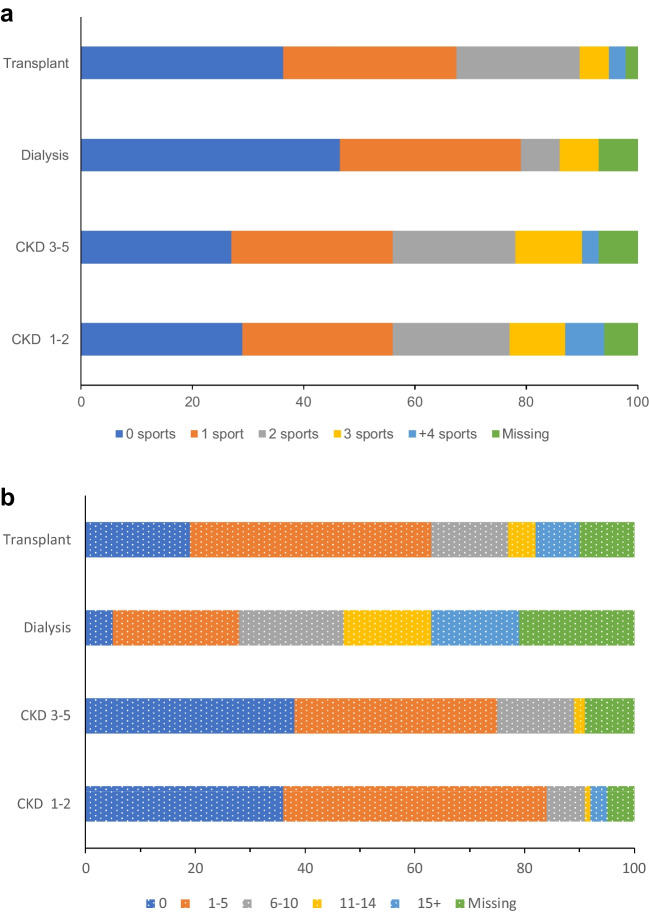
**a** Frequency of sport activities and participation across CKD stages. **b** Number days of school absence over a 4-week period across CKD stages

**Fig. 3:**
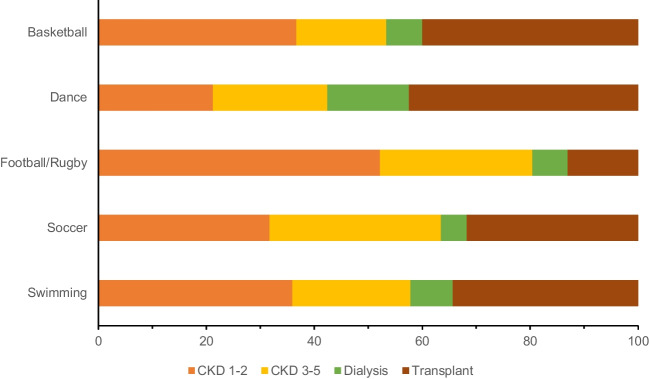
Popular sport activities played by children with CKD, stratified by CKD stage

Figure 2a illustrates the frequency of sports participation stratified by stages of CKD. Approximately 50% of children treated with dialysis played no sports, compared to 36% of kidney transplant recipients, 25% of children with CKD 3–5, and 27% of children with CKD 1–2. Of those who participated in sporting activities, only 7% of children treated with dialysis played more than two types of sports, compared to 20% for those with mild to moderate stage CKD (CKD 1–2 and 3–5). Figure 3 illustrates the five most common sports played by children with CKD, stratified by CKD stages. Overall, the most popular sports played amongst children with CKD were swimming (17%), soccer (17%), football/rugby (12%), dance (9%), and basketball (8%). Participation in football/rugby was higher amongst children with CKD 1–2 (52%) and CKD 3–5 (28%) than children treated with dialysis (7%) and with kidney transplants (13%) (Fig. 3). Compared to children with CKD 1–2, the incidence rate ratio (IRR) (95%CI) for sports participation amongst children with CKD 3–5, dialysis, and transplant were 0.84 (0.64–1.09), 0.59 (0.39–0.90), and 0.75 (0.58–0.96), respectively, adjusted for SES and ethnicity (Table 1) (full model results in Supplemental Appendix Table 3). Similar results were obtained when a negative binomial model was fitted instead of a Poisson model.

**Table 1:** Association between CKD stage and the number of sports played by children with CKD

		Univariable		Multivariable*	
Covariate		IRR (95% CI)	*p* value	IRR (95% CI)	*p* value
CKD stage			< 0.01		0.04
	CKD 1–2	Ref		Ref	
	CKD 3–5	0.93 (0.73–1.20)		0.84 (0.64–1.09)	
	Dialysis	0.52 (0.35–0.77)		0.59 (0.39–0.90)	
	Transplant	0.77 (0.61–0.97)		0.75 (0.58–0.96)	

*CKD* chronic kidney disease, *IRR* incidence rate ratio, *95% CI* 95% confidence interval

*Adjusted for socioeconomic status and ethnicity

### Association between CKD stage and school absences

**Fig. 4:**
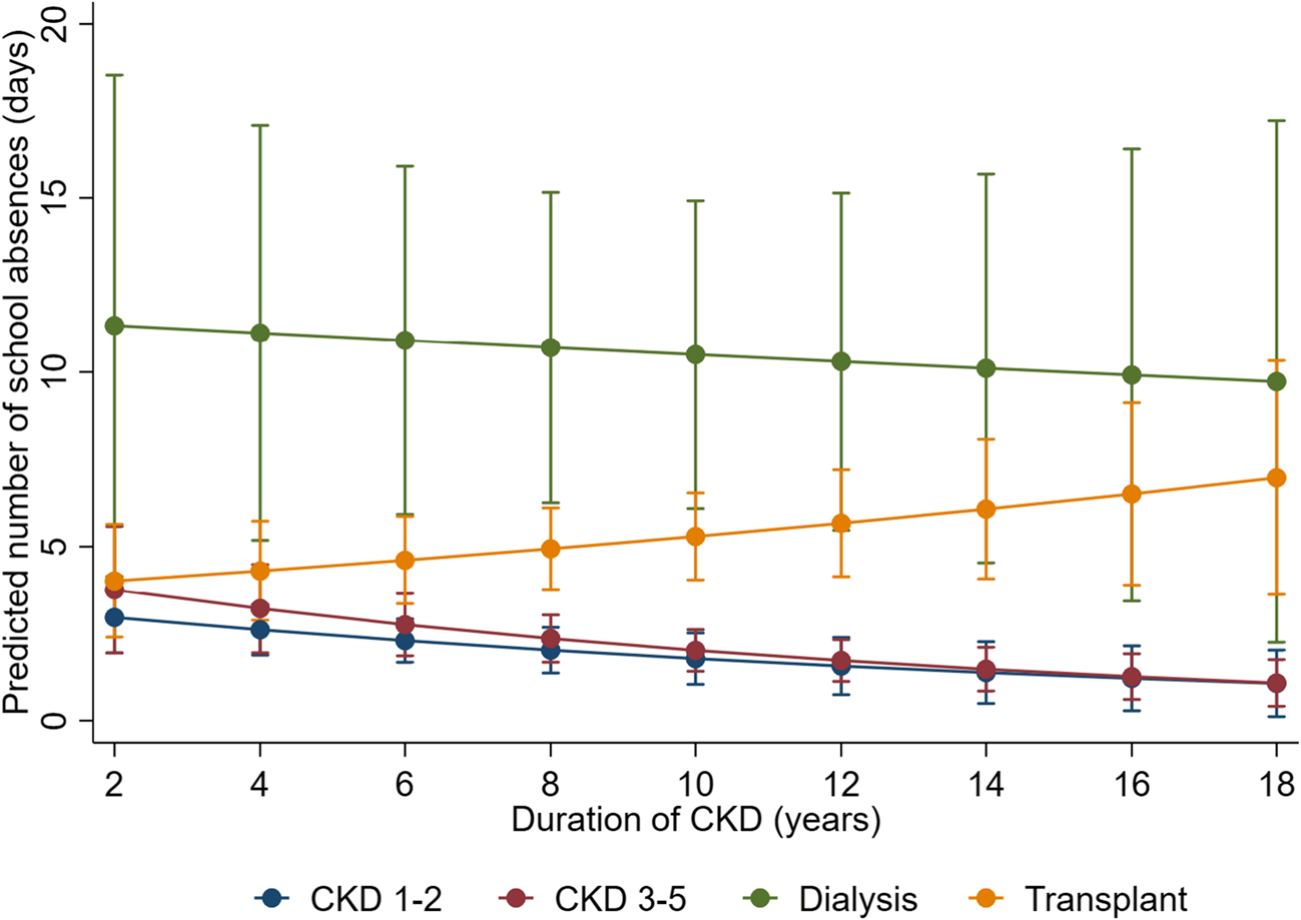
Modification of effect of CKD stage on school absences by duration of CKD

Figure 2b shows the frequency of school absences by CKD stage. Children and adolescents treated with dialysis had the highest number of school absences over a four-week period (median: 9 days, IQR: 5–15 days), followed by transplant recipients (median: 2 days, IQR: 1–7 days), children with CKD 3–5 (median: 1 days, IQR: 0–3 days), and children with CKD 1–2 (median: 1 days, IQR: 0–3 days). After adjusting for ethnicity, age, SES, and CKD cause, we found the association of CKD stage with number of school absences over a four-week period to be modified by the duration of CKD diagnosis (*p*-interaction < 0.01). Results of the predicted number of school days absent using marginal means is presented in Table 2. For children with CKD 1–2, CKD 3–5, and on dialysis, increasing duration of CKD was associated with reduced number of school absences. When duration of CKD was four years, the number of school days absent was 2.6 days amongst children with CKD 1–2, decreasing to 1.2 days when duration of CKD was 16 years with a similar number of absences seen in children with CKD 3–5 (Table 2). Amongst children on dialysis, the number of school days missed was 11 days when duration of CKD was 4 years, decreasing to 9.9 days when duration of CKD was 16 years (Table 2). In contrast, increasing duration of CKD was associated with a higher number of school absences amongst children with a kidney transplant. Amongst children with a kidney transplant, the number of school days missed for children with a kidney transplant was 4.3 days when duration of CKD was 4 years, increasing to 6.5 days with duration of CKD was 16 years (Table 2). Figure 4 provides graphical representation of this interaction through the predicted number of school days missed over duration of CKD, stratified by CKD stage with full model results presented in Supplemental Appendix Table 4. We did not identify evidence of effect modification of CKD stage by SES, age, ethnicity, or CKD cause (*p*-interaction > 0.01). We conducted a sensitivity analysis of our results excluding the 34 participants in the study who were within 12 months since their kidney transplant and found comparable effect of CKD stage on school attendance. Within the sensitivity analysis, CKD stage continued to modify the effect of duration of CKD on school absence as seen in the primary analysis (*p*-interaction 0.05). We also investigated the correlation between number of school days absent and total number of sports played and found increasing days absent was associated with lower number of sports played (Spearman’s rho − 0.13, *p* = 0.02).

**Table 2:** Interactive effect of CKD stage and duration of CKD on days of school absences

Duration of CKD	Number of school days missed (95% CI)*			
	CKD 1–2	CKD 3–5	Dialysis	Transplant
4 years	2.6 (1.87–3.33)	3.2 (1.94–4.48)	11.1 (5.18–17.07)	4.30 (2.88–5.73)
8 years	2.0 (1.36–2.67)	2.4 (1.67–3.03)	10.7 (6.26–15.16)	4.9 (3.77–6.11)
12 years	1.6 (0.74–2.38)	1.7 (1.12–2.32)	10.3 (5.47–15.14)	5.7 (4.14–7.20)
16 years	1.2 (0.28–2.14)	1.3 (0.61–1.91)	9.9 (3.43–16.40)	6.5 (3.90–9.12)

*CKD* chronic kidney disease, *95% CI* 95% confidence interval

*Adjusted for ethnicity, socioeconomic status, age, and CKD cause. The predicted number of school days absent over a four-week period using marginal means is presented

## Discussion

In our large binational cohort of children with CKD, we found a direct association between CKD stage, school absences, and sport participation. Children treated with KRT were less likely to participate in sport activities, with children on dialysis and with kidney transplants participating in approximately 40% and 25% fewer sports than children with mild to moderate stage CKD, respectively. The impact of CKD stage on school attendance was modified by the duration of CKD diagnoses, with children treated with KRT who had a prolonged duration of CKD diagnosis reporting more days of school absences compared to their peers with shorter duration of CKD diagnosis.

The observed findings for sport reflect reduced levels of physical activity seen in children with CKD, particularly amongst children with advanced stage kidney disease. A cross-sectional survey of children with kidney transplants found lower levels of physical activity compared to their peers, with only 30% participating in organised sport in the past year [14]. Children with kidney transplants also took fewer steps per day and fewer minutes per day engaged in physical activity compared to children without CKD [15]. Other studies have also found lower levels of physical activity in children with earlier stages of CKD. In the United States, only 13.4% of children with CKD 3–5 met the national weekly physical activity recommendations of 60 min of physical activity daily for seven days compared to 25% of children in the general population. Additionally, the median number of team sports played in the past 12 months was one (IQR 0–2) which was similar to the median number of sports played by the participants in our cohort [10].

The sports played amongst children with CKD mirrored the popular sports played by school-aged children in the general population in Australia and New Zealand where the most popular sports are swimming, soccer, rugby/football, dance, and basketball [16, 17]. Aside from rugby/football which was played less frequently amongst children with a kidney transplant, the types of sports engagement did not differ by CKD stages. Fears about transplant kidney injury were the predominant reason for limitation in physical activity listed by children with a kidney transplant in one single centre study, where two thirds of children received counselling about restricting contact sports such as rugby and touch football [14]. This concern likely accounts for the lower participation rates of rugby/football amongst transplant recipients in our study. Whilst sport participation is reduced amongst children with CKD, particularly for those treated with KRT, our study illustrates that the types of sports played are relatively similar to the general population [16, 17].

There are many reasons for reduced sports participation amongst children on dialysis or with a kidney transplant. Prior studies have found cardiorespiratory fitness, as measured by peak oxygen consumption and muscle strength, in children on dialysis was substantially below the normal range when compared to their age-matched peers [18, 19]. Although studies have found some improvement in cardiorespiratory fitness post-transplant, it remains poorer when compared to the general population [18]. Other barriers to sport participation may include lower perceptions of sport competence amongst kidney transplant recipients, symptoms such as fatigue, medical treatments such as placement of dialysis catheters, and also concerns about potential damage to the kidney transplant [8, 15]. Patients also report issues around self-esteem, and social anxiety about peer judgement also contribute to reduced participation in sport. We hypothesise that cost constraints also likely contribute to reduced sport participation for children receiving KRT, as well as reduced time for extracurricular activities due to demands from medical treatments and attendance at hospital, and finally parental anxiety about possible injury may contribute.

Poor school attendance amongst children with CKD has been previously characterised, with 17% of children with CKD stages 1–4 missing greater than 18 days of school a year in North America, compared to 3% of the general population [9]. School absenteeism remains higher amongst children post-kidney transplant, with a mean school attendance of 85%, compared to 94% amongst peers. Frequent hospital attendance and admission were considered the major contributors to school absences [20]. We found a differential effect of CKD stage on school attendance, based on the duration of CKD. The duration of reduced kidney function over time had a profound impact on the ability to attend school. For children with kidney transplants and on dialysis, the number of missed school days increased with longer duration of CKD diagnoses. Children with CKD 1–2 missed less school if they had CKD for a longer period of time, suggesting adaptation over time. In contrast, school absences in children on dialysis were high, with little change with duration of CKD. Although children with a kidney transplant did not appear to miss more school compared to children with CKD 1–2, the amount of school they missed increased with longer duration of CKD. Taken overall, we hypothesise this suggests a cumulative effect of chronic kidney disease amongst those who progress to kidney failure. Ongoing and augmented support is needed to improve school attendance such as enhanced school liaison, assistance with transport and minimisation, and coordination of hospital visits. Previous studies have shown chronic school absence in children with CKD 1–4 is associated with enuresis and needing catheterisation, higher medication burden, hospitalisation, and presentation to the emergency department [9]. Reasons for school absenteeism reported by children with CKD stages 1–4 and their caregivers include feeling chronically unwell, having a relentless number of medical appointments, and being bullied by other students at school and extra-curricular activities [21]. Qualitative studies also suggest fatigue, illness, discomfort, and medical treatments as contributors to poor school attendance [8]. Future research is necessary to comprehensively assess the reasons behind reduced school attendance in children across all stages of CKD, which will help identify strategies to improve school attendance.

Our study has several strengths. We have, for the first time, evaluated the two key aspects of life participation in a large cohort of children with CKD spanning all stages of CKD through to KRT. Our cohort is broadly representative of school-age children with CKD in Australia and New Zealand, with representation from diverse ethnic groups and across levels of SES. Our study also has several limitations. We have not captured data on preschool-aged children and acknowledge that reduced life participation may be evident even in early childhood in children with CKD. Our measure of school absenteeism is restricted to the four weeks prior to when the questionnaire was undertaken and could be skewed by a single prolonged illness or hospitalisation. Furthermore, comparison to a general population measure of school attendance rate over a calendar year is limited. The interpretation of days of school absent was left to parents and carers, and we are unable to differentiate between full or partial day absences for children in the study. There was a higher proportion of missing school attendance data amongst children on dialysis, with comments suggesting that this was due to having high levels of school absence. This may mean we have underestimated the effect of dialysis on school attendance. We did not have granular data on physical activity such as minutes and steps per day or the setting and intensity of participation in sport. We also did not have comparative data on sport participation in children without CKD. We did not collect data on parent and caregiver participation in sport, and acknowledge this may have had a confounding effect on child sport participation. We also did not specify a time frame for sport participation in our questionnaire. Children treated with dialysis may experience more interruptions in sports activities, whilst those with early to moderate stage CKD and with stable transplants may participate in sports activities (irrespective of the types of sports) more consistently because they are less burdened by their illnesses. The other important domain of life participation identified by patients and their families was the ability to participate in social activities [8]. Whilst we asked broadly about hobbies in our questionnaires, we did not collect information about their ability to interact socially and capacity to keep up with their peers without CKD. This is important as to be fully engaged in all aspects of life participation and to have optimal psychological wellbeing the ability to form meaningful social relationships is critical. Furthermore, participation in other hobbies and activities may reduce children’s participation in sport and may partially account for the lower rates of sport participation seen in children on dialysis and with a kidney transplant.

In conclusion, findings from this large observational study highlight that life participation, evaluated by the number and types of sports played and the number of school absences, is significantly reduced amongst children with CKD, and the influence of CKD is heightened considerably amongst children treated with KRT. Children with CKD face immense challenges associated with their illness affecting all aspects of their daily lives. Healthcare professionals and policy makers may have neglected this crucial element of a child’s life with CKD. Further research is warranted to identify the barriers and solutions to improve life participation and engagement in our children with CKD. Active collaborations between healthcare providers, caregivers, and schools are needed to ensure our children have opportunities to thrive and participate in life more fully.

### Supplementary Information

Below is the link to the electronic supplementary material.

Supplementary file1 (DOCX 43.9 KB)

Graphical abstract (PPTX 54.8 KB)

## Data Availability

The data that support the findings of this study is restricted as due to the ethics approval for this study, and are not publicly available.
