# Supplementary material for: School attendance and sport participation amongst children with chronic kidney disease: a cross-sectional analysis from the Kids with CKD (KCAD) study
Source: Pediatr Nephrol. 2023 Nov 9;39(4):1229–37. doi: 10.1007/s00467-023-06198-0 (PMC10899305; doi:10.1007/s00467-023-06198-0)
Supplement: Supplementary file 1 — Supplementary file1 (DOCX 43.9 KB) [file 467_2023_6198_MOESM1_ESM.docx]

**Supplementary Table 1. Baseline characteristics of the study cohort**

|  | | | | | |  |
| --- | --- | --- | --- | --- | --- | --- |
|  | **CKD stage 1-2 (n=107)** | **CKD stage 3-5 (n=92)** | **Dialysis**  **(n=43)** | **Transplant**  **(n=136)** | **All**  **(n=377)** | |
| **Gender (n(%))** |  |  |  |  |  | |
| Male | 67 (63) | 66 (72) | 21 (49) | 79 (59) | 233 (62) | |
| Female | 40 (37) | 26 (28) | 22 (51) | 56 (41) | 144 (38) | |
| **Age (mean (SD))** | 11.8 (3.5) | 11.7 (3.7) | 12.9 (3.7) | 12.5 (4.0) | 12.2 (3.8) | |
| **Underlying Disease (n(%))** |  |  |  |  |  | |
| CAKUT | 12 (11) | 50 (54) | 18 (42) | 47 (35) | 127 (34) | |
| Nephrotic | 55 (51) | 4 (4) | 6 (14) | 27 (20) | 92 (24) | |
| Glomerulonephritis | 27 (25) | 7 (8) | 6 (14) | 18 (13) | 58 (15) | |
| Cystic | 1 (1) | 13 (14) | 4 (9) | 13 (10) | 31 (8) | |
| Other | 12 (11) | 18 (20) | 9 (21) | 30 (22) | 69 (18) | |
| **Ethnicity (n(%)) (n=370)** |  |  |  |  |  | |
| European | 52 (49) | 56 (61) | 24 (56) | 88 (65) | 220 (58) | |
| Asian | 32 (30) | 8 (9) | 2 (5) | 16 (12) | 58 (15) | |
| Middle Eastern | 8 (7) | 14 (15) | 7 (16) | 13 (10) | 42 (11) | |
| Other | 12 (11) | 14 (15) | 9 (21) | 15 (11) | 50 (13) | |
| **BMI (z-score) (mean (SD) (n=369)** | 0.41 (1.43) | 0.13 (1.32) | 0.34 (1.32) | 0.67 (1.15) | 0.42 (1.30) | |
| Normal/Thinness (< 85^th^ centile) | 69 (64) | 66 (72) | 29 (67) | 82 (61) | 246 (65) | |
| Overweight (≥ 85^th^ centile) | 22 (21) | 18 (20) | 8 (19) | 35 (26) | 83 (22) | |
| Obesity (≥ 95^th^ centile) | 14 (13) | 6 (7) | 4 (9) | 16 (12) | 40 (11) | |
| **Private Health Insurance (n(%)) (n=376)** | 35 (33) | 43 (47) | 7 (16) | 50 (37) | 135 (36) | |
| **Time since Diagnosis (Years), (mean (SD))(n=376)** | 5.8 (4.7) | 8.9 (4.7) | 9.6 (5.9) | 9.2 (4.8) | 8.2 (5.1) | |
| **Global family socioeconomic status (n=354)** |  |  |  |  |  | |
| <25^th^ Percentile | 16 (15) | 26 (28) | 17 (40) | 27 (20) | 86 (23) | |
| 25^th^ – 50^th^ Percentile | 30 (28) | 17 (18) | 10 (23) | 36 (27) | 93 (25) | |
| 50^th^ – 75^th^ Percentile | 28 (26) | 22 (24) | 7 (16) | 33 (24) | 90 (24) | |
| >75^th^ Percentile | 26 (24) | 25 (27) | 4 (9) | 30 (22) | 85 (23) | |
| **Education (n=368)** |  |  |  |  |  | |
| Local/other school | 97 (99) | 96 (98) | 38 (97) | 129 (96) | 360 (98) | |
| Home/distance education | 1 (1) | 2 (2) | 1 (3) | 4 (3) | 8 (2) | |

**Supplementary Table 2. Total number of sports played and number of school absences over a 4-week period across CKD stage**

|  | CKD 1-2 | CKD 3-5 | Dialysis | Transplant | All |
| --- | --- | --- | --- | --- | --- |
|  | n = 107  n (%) | n = 92  n (%) | n = 43  n (%) | n = 135  n (%) | n = 377  n (%) |
| **Total sports played** |  |  |  |  |  |
| 0 | 31 (29) | 25 (27) | 20 (47) | 49 (36) | 125 (33) |
| 1 | 29 (27) | 27 (29) | 14 (33) | 42 (31) | 112 (30) |
| 2 | 23 (21) | 20 (22) | 3 (7) | 30 (22) | 76 (20) |
| 3 | 11 (10) | 11 (12) | 3 (7) | 7 (5) | 32 (8) |
| 4+ | 7 (7) | 3 (3) | 0 (0) | 4 (3) | 14 (4) |
| Missing | 6 (6) | 6 (7) | 3 (7) | 3 (2) | 18 (5) |
| **School days missed** |  |  |  |  |  |
| 0 | 39 (36) | 35 (38) | 2 (5) | 25 (19) | 101 (27) |
| 1-5 | 51 (48) | 34 (37) | 10 (23) | 59 (44) | 154 (41) |
| 6-10 | 8 (7) | 13 (14) | 8 (19) | 19 (14) | 48 (13) |
| 11-15 | 1 (1) | 2 (2) | 7 (16) | 7 (5) | 17 (5) |
| 15+ | 3 (3) | 0 (0) | 7 (16) | 11 (8) | 21 (6) |
| Missing | 5 (5) | 8 (9) | 9 (21) | 14 (10) | 36 (10) |

**Supplemental Table 3: Univariate and multivariable model results for sport participation**

|  |  | Univariable | | Multivariable |  |
| --- | --- | --- | --- | --- | --- |
| **Covariate** |  | IRR (95% CI) | p value | IRR (95% CI) | p value |
| **CKD Stage** |  |  | < 0.01 |  | 0.04 |
|  | CKD 1-2 | Ref |  | Ref |  |
|  | CKD 3-5 | 0.93 (0.73-1.20) |  | 0.84 (0.64-1.09) |  |
|  | Dialysis | 0.52 (0.35-0.77) |  | 0.59 (0.39-0.90) |  |
|  | Transplant | 0.77 (0.61-0.97) |  | 0.75 (0.58-0.96) |  |
| **Ethnicity** |  |  | 0.02 |  | 0.05 |
|  | European | Ref |  | Ref |  |
|  | Asian | 0.68 (0.50-0.93) |  | 0.67 (0.48-0.94) |  |
|  | Middle Eastern | 0.65 (0.46-0.93) |  | 0.80 (0.56-1.16) |  |
|  | Other | 0.91 (0.69-1.22) |  | 1.12 (0.83-1.52) |  |
| **Socioeconomic Status** |  |  | < 0.01 |  | <0.01 |
|  | <25^th^ Percentile | 0.43 (0.32-0.58) |  | 0.48 (0.35-0.65) |  |
|  | 25^th^-50^th^ Percentile | 0.47 (0.36-0.62) |  | 0.50 (0.38-0.67) |  |
|  | 50^th^-75^th^ Percentile | 0.71 (0.56-0.91) |  | 0.73 (0.57-0.93) |  |
|  | >75^th^ Percentile | Ref |  | Ref |  |
| **Gender** |  |  | <0.01 |  |  |
|  | Male | Ref |  |  |  |
|  | Female | 0.74 (0.61-0.91) |  |  |  |
| **Age** | One year increase | 0.97 (0.94-0.99) | 0.01 |  |  |
| **Private Health Insurance** |  |  | <0.01 |  |  |
|  | No | Ref |  |  |  |
|  | Yes | 1.31 (1.08-1.59) |  |  |  |
| **Duration of CKD** | One year increase | 0.98 (0.97-1.00) | 0.09 |  |  |
| **CKD Cause** |  |  | 0.44 |  |  |
|  | CAKUT | Ref |  |  |  |
|  | Cystic | 1.18 (0.92-1.52) |  |  |  |
|  | Glomerulonephritis | 1.01 (0.75-1.37) |  |  |  |
|  | Nephrotic | 0.86 (0.56-1.26) |  |  |  |
|  | Other | 1.12 (0.85-1.46) |  |  |  |

**Supplemental Table 4: Association between CKD stage and school absenteeism**

|  |  | Univariable | | Multivariable |  |
| --- | --- | --- | --- | --- | --- |
| **Covariate** |  | IRR (95% CI) | p value | IRR (95% CI) | p value |
| **CKD Stage** |  |  | <0.01 |  | 0.12 |
|  | CKD 1-2 | Ref |  | Ref |  |
|  | CKD 3-5 | 1.05 (0.70-1.55) |  | 1.29 (0.62-2.67) |  |
|  | Dialysis | 4.32 (2.62-7.10) |  | 2.77 (1.18-6.52) |  |
|  | Transplant | 2.26 (1.59-3.22) |  | 0.99 (0.52-1.89) |  |
| **Ethnicity** |  |  | <0.01 |  | <0.01 |
|  | European | Ref |  | Ref |  |
|  | Asian | 0.59 (0.38-0.91) |  | 0.56 (0.37-0.86) |  |
|  | Middle Eastern | 1.38 (0.85-2.23) |  | 1.06 (0.67-1.68) |  |
|  | Other | 1.62 (1.03-2.54) |  | 1.68 (1.12-2.53) |  |
| **Socioeconomic Status** |  |  | 0.03 |  | 0.03 |
|  | <25^th^ | 1.89 (1.21-2.96) |  | 1.86 (1.23-2.83) |  |
|  | 25^th^-50^th^ | 1.70 (1.10-2.64) |  | 1.50 (0.99-2.28) |  |
|  | 50^th^-75^th^ | 1.54 (0.99-2.40) |  | 1.29 (0.85-1.95) |  |
|  | >75^th^ | Ref |  | Ref |  |
| **Gender** |  |  | 0.08 |  |  |
|  | Male | Ref |  |  |  |
|  | Female | 1.32 (0.97-1.79) |  |  |  |
| **Age** | One year increase | 1.04 (1.-1.08) | 0.07 | 1.05 (1.00 – 1.10) | 0.04 |
| **Private Health Insurance** |  |  | 0.03 |  |  |
|  | No | Ref |  |  |  |
|  | Yes | 0.84 (0.61-1.16) |  |  |  |
| **Duration of CKD (CKD stage 1-2)** | One year increase | 1.00 (0.97-1.03) | 0.93 | 0.94 (0.88 – 1.00) | 0.06 |
| **CKD Cause** |  |  | 0.29 |  | <0.01 |
|  | CAKUT | Ref |  | Ref |  |
|  | Cystic | 1.20 (0.80-1.81) |  | 2.37 (1.41-3.99) |  |
|  | Glomerulonephritis | 1.37 (0.87-2.17) |  | 1.58 (0.98-2.53) |  |
|  | Nephrotic | 1.65 (0.94-2.89) |  | 1.98 (1.29-3.05) |  |
|  | Other | 1.47 (0.94-2.28) |  | 1.64 (1.08-2.45) |  |
| **CKD stage x duration of CKD** | Per one year increase duration of CKD |  |  |  | <0.01 |
|  | CKD 3-5 |  |  | 0.99 (0.90-1.07) |  |
|  | Dialysis |  |  | 1.06 (0.96-1.16) |  |
|  | Transplant |  |  | 1.10 (1.02-1.19) |  |

**Strobe Checklist**

STROBE Statement—Checklist of items that should be included in reports of ***cross-sectional studies***

|  | Item No | Recommendation | Checked? |
| --- | --- | --- | --- |
| **Title and abstract** | 1 | (*a*) Indicate the study’s design with a commonly used term in the title or the abstract | 1 |
|  |  | (*b*) Provide in the abstract an informative and balanced summary of what was done and what was found | 2 |
| Introduction | | |  |
| Background/rationale | 2 | Explain the scientific background and rationale for the investigation being reported | 3 |
| Objectives | 3 | State specific objectives, including any prespecified hypotheses | 3 |
| Methods | | |  |
| Study design | 4 | Present key elements of study design early in the paper | 4 |
| Setting | 5 | Describe the setting, locations, and relevant dates, including periods of recruitment, exposure, follow-up, and data collection | 4 |
| Participants | 6 | (*a*) Give the eligibility criteria, and the sources and methods of selection of participants | 4 |
| Variables | 7 | Clearly define all outcomes, exposures, predictors, potential confounders, and effect modifiers. Give diagnostic criteria, if applicable | 4-5 |
| Data sources/ measurement | 8* | For each variable of interest, give sources of data and details of methods of assessment (measurement). Describe comparability of assessment methods if there is more than one group | *4-5* |
| Bias | 9 | Describe any efforts to address potential sources of bias | 4-5 |
| Study size | 10 | Explain how the study size was arrived at | NA |
| Quantitative variables | 11 | Explain how quantitative variables were handled in the analyses. If applicable, describe which groupings were chosen and why | 5-6 |
| Statistical methods | 12 | (*a*) Describe all statistical methods, including those used to control for confounding | 5-6 |
|  |  | (*b*) Describe any methods used to examine subgroups and interactions | 5-6 |
|  |  | (*c*) Explain how missing data were addressed | 5-6 |
|  |  | (*d*) If applicable, describe analytical methods taking account of sampling strategy | NA |
|  |  | (*e*) Describe any sensitivity analyses | NA |
| Results | | |  |
| Participants | 13* | (a) Report numbers of individuals at each stage of study—eg numbers potentially eligible, examined for eligibility, confirmed eligible, included in the study, completing follow-up, and analysed | 16 |
|  |  | (b) Give reasons for non-participation at each stage | 16 |
|  |  | (c) Consider use of a flow diagram | 16 |
| Descriptive data | 14* | (a) Give characteristics of study participants (eg demographic, clinical, social) and information on exposures and potential confounders | Supp |
|  |  | (b) Indicate number of participants with missing data for each variable of interest | Supp |
| Outcome data | 15* | Report numbers of outcome events or summary measures | 6 |
| Main results | 16 | (*a*) Give unadjusted estimates and, if applicable, confounder-adjusted estimates and their precision (eg, 95% confidence interval). Make clear which confounders were adjusted for and why they were included | 6-7 |
|  |  | (*b*) Report category boundaries when continuous variables were categorized | 5-6 |
|  |  | (*c*) If relevant, consider translating estimates of relative risk into absolute risk for a meaningful time period | NA |
| Other analyses | 17 | Report other analyses done—eg analyses of subgroups and interactions, and sensitivity analyses | 6-7 |
| Discussion | | |  |
| Key results | 18 | Summarise key results with reference to study objectives | 7-10 |
| Limitations | 19 | Discuss limitations of the study, taking into account sources of potential bias or imprecision. Discuss both direction and magnitude of any potential bias | 10 |
| Interpretation | 20 | Give a cautious overall interpretation of results considering objectives, limitations, multiplicity of analyses, results from similar studies, and other relevant evidence | 7-10 |
| Generalisability | 21 | Discuss the generalisability (external validity) of the study results | 8-10 |
| Other information | | |  |
| Funding | 22 | Give the source of funding and the role of the funders for the present study and, if applicable, for the original study on which the present article is based | 11 |

*Give information separately for exposed and unexposed groups.
