# Supplementary material for: School attendance and sport participation amongst children with chronic kidney disease: a cross-sectional analysis from the Kids with CKD (KCAD) study
Source: Pediatr Nephrol. 2023 Nov 9;39(4):1229–37. doi: 10.1007/s00467-023-06198-0 (PMC10899305; doi:10.1007/s00467-023-06198-0)
Supplement: Supplementary file 2 — Graphical abstract (PPTX 54.8 KB) [file 467_2023_6198_MOESM2_ESM.pptx]

## Slide 1
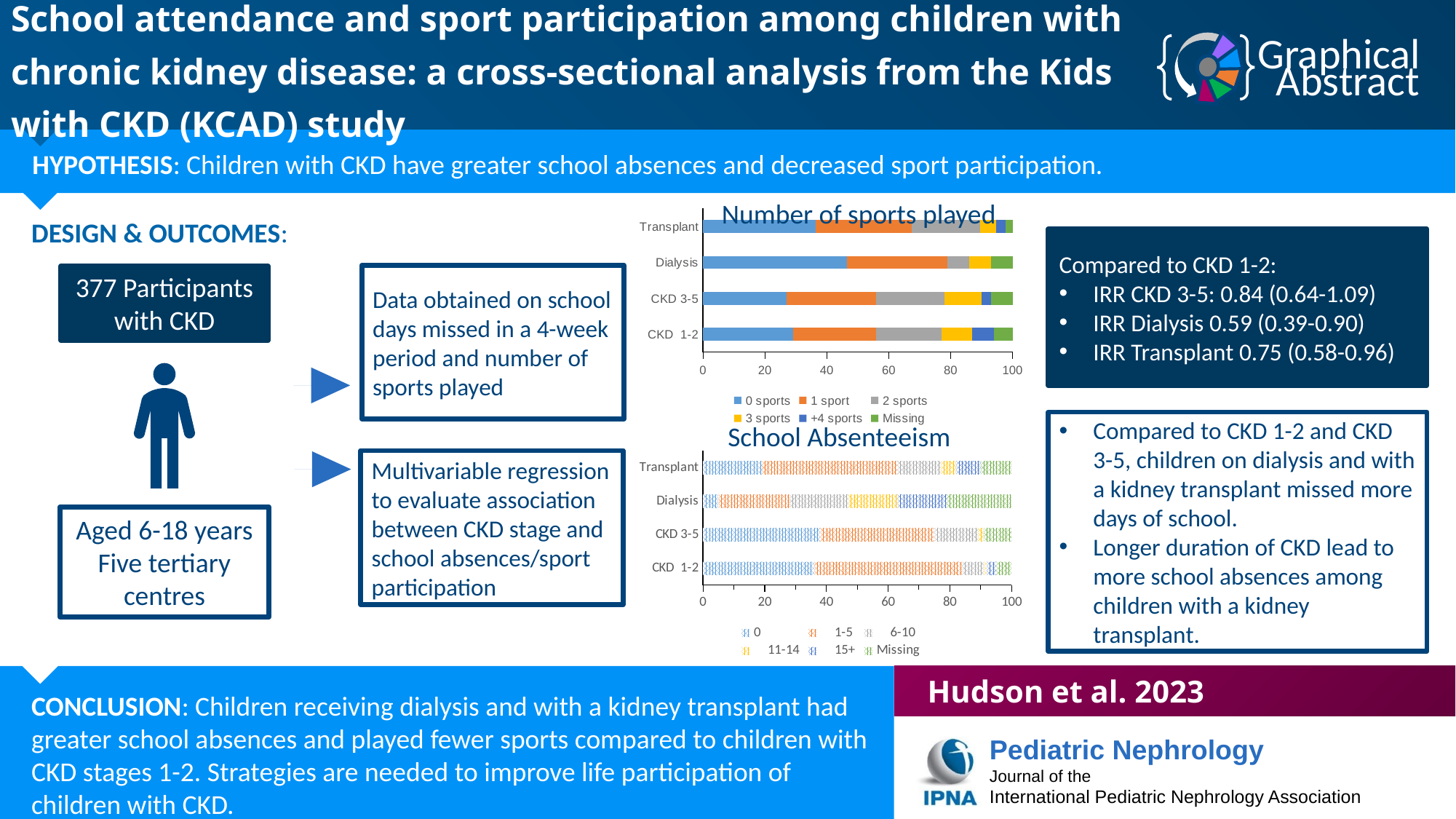

School attendance and sport participation among children with chronic kidney disease: a cross-sectional analysis from the Kids with CKD (KCAD) study
HYPOTHESIS: Children with CKD have greater school absences and decreased sport participation.
Number of sports played
### Chart
| Category | | | | | | |
|---|---|---|---|---|---|---|
| CKD  1-2 | 29.0 | 27.0 | 21.0 | 10.0 | 7.0 | 6.0 |
| CKD 3-5 | 27.0 | 29.0 | 22.0 | 12.0 | 3.0 | 7.0 |
| Dialysis | 46.5 | 32.5 | 7.0 | 7.0 | 0.0 | 7.0 |
| Transplant | 36.3 | 31.1 | 22.2 | 5.2 | 3.0 | 2.2 |DESIGN & OUTCOMES:
Compared to CKD 1-2:
IRR CKD 3-5: 0.84 (0.64-1.09)
IRR Dialysis 0.59 (0.39-0.90)
IRR Transplant 0.75 (0.58-0.96)
Data obtained on school days missed in a 4-week period and number of sports played
Multivariable regression to evaluate association between CKD stage and school absences/sport participation
377 Participants with CKD
Aged 6-18 years Five tertiary centres
Compared to CKD 1-2 and CKD 3-5, children on dialysis and with a kidney transplant missed more days of school.
Longer duration of CKD lead to more school absences among children with a kidney transplant.
School Absenteeism
### Chart
| Category | 0 | 1-5 | 6-10 | 11-14 | 15+ | Missing |
|---|---|---|---|---|---|---|
| CKD  1-2 | 36.0 | 48.0 | 7.0 | 1.0 | 3.0 | 5.0 |
| CKD 3-5 | 38.0 | 37.0 | 14.0 | 2.0 | 0.0 | 9.0 |
| Dialysis | 5.0 | 23.0 | 19.0 | 16.0 | 16.0 | 21.0 |
| Transplant | 19.0 | 44.0 | 14.0 | 5.0 | 8.0 | 10.0 |Hudson et al. 2023
CONCLUSION: Children receiving dialysis and with a kidney transplant had greater school absences and played fewer sports compared to children with CKD stages 1-2. Strategies are needed to improve life participation of children with CKD.
